# Supplementary material for: Effective screening strategies for safe opening of universities under Omicron and Delta variants of COVID-19
Source: Sci Rep. 2022 Dec 9;12:21309. doi: 10.1038/s41598-022-25801-2 (PMC9734754; doi:10.1038/s41598-022-25801-2)
Supplement: Supplementary file 1 — Supplementary Information. [file 41598_2022_25801_MOESM1_ESM.pdf]

Supplementary Information for "Effective screening strategies for safe opening of universities under Omicron and Delta variants of COVID-19"

Marie Jeanne Rabil, Sait Tunc, Douglas R. Bish, Ebru K. Bish

Model Description

We developed an extended SEIR (Susceptible, Exposed, Infectious, Removed) framework to model COVID-19 infection spread in a heterogeneous hypothetical population, comprised of faculty and student groups, considering protective and preventative interventions including screening, isolation, masking, and vaccination, when two virus variants are in circulation. The model is a deterministic epidemic model, and tracks the individuals as they transition through different health states (compartments), and the overall flow is governed by a series of difference equations.

In particular, our compartmental model expands that in [9], to consider the following distinct features, see also the flow-chart in Fig. S1:

- We model both vaccine-induced immunity and infection-induced (natural) immunity, that is, an individual can develop protection against a future infection due to either prior vaccination or a prior COVID-19 infection. In particular:
  - We model variant- and dose-dependent vaccine effectiveness. Each individual can be in one of the following compartments based on their vaccination status: "unvaccinated," "vaccinated" (fully vaccinated either with a 1-dose or a 2-dose vaccine prior to August 2021), and "boosted" (fully vaccinated plus received the

booster in January 2022). Because the vaccinated individuals (i.e., without a booster) are assumed to receive their last dose by the start of Fall 2021 semester, we also model waning immunity.

  - We model natural immunity for those individuals who have recovered from a prior infection (through the compartments, recovered & unknown, and recovered & known).

- We model population-based disparities in disease spread, hospitalization, and mortality rates to consider student versus and faculty groups, and their vaccination status. We also consider that the effectiveness of the vaccine depends on whether an individual has received the booster or not, as well as on the circulating virus variant.

- We model variant- and group-dependent disease transmission rates.

In addition, our model preserves the following features from [9]:

- The test result becomes available 8 hours after taking the test. A positive test result indicates either a "false-positive," or "asymptomatic & infected;" and any subject with a positive test result moves to isolation as soon as they receive their test result. No transmission can occur during isolation. All false-positives are corrected the next day. Both the "asymptomatic & infected" and "symptomatic & infected" subjects have an isolation time with a mean of 5 days, after which they move to the "knowingly immune" compartment, unless they are hospitalized. We assume ample isolation capacity and perfect compliance with isolation orders.

- A subject can receive natural immunity through a prior infection; this is modeled through two compartments: The subjects in the "knowingly immune" compartment have gained natural immunity through a prior asymptomatic infection that was detected during routine screening, or a prior symptomatic infection (leading to isolation in each case). The subjects in the "recovered & unknown" compartment have gained

natural immunity through a prior asymptomatic infection that was undetected, thus, these subjects cannot be differentiated from "uninfected" individuals, and might get tested.

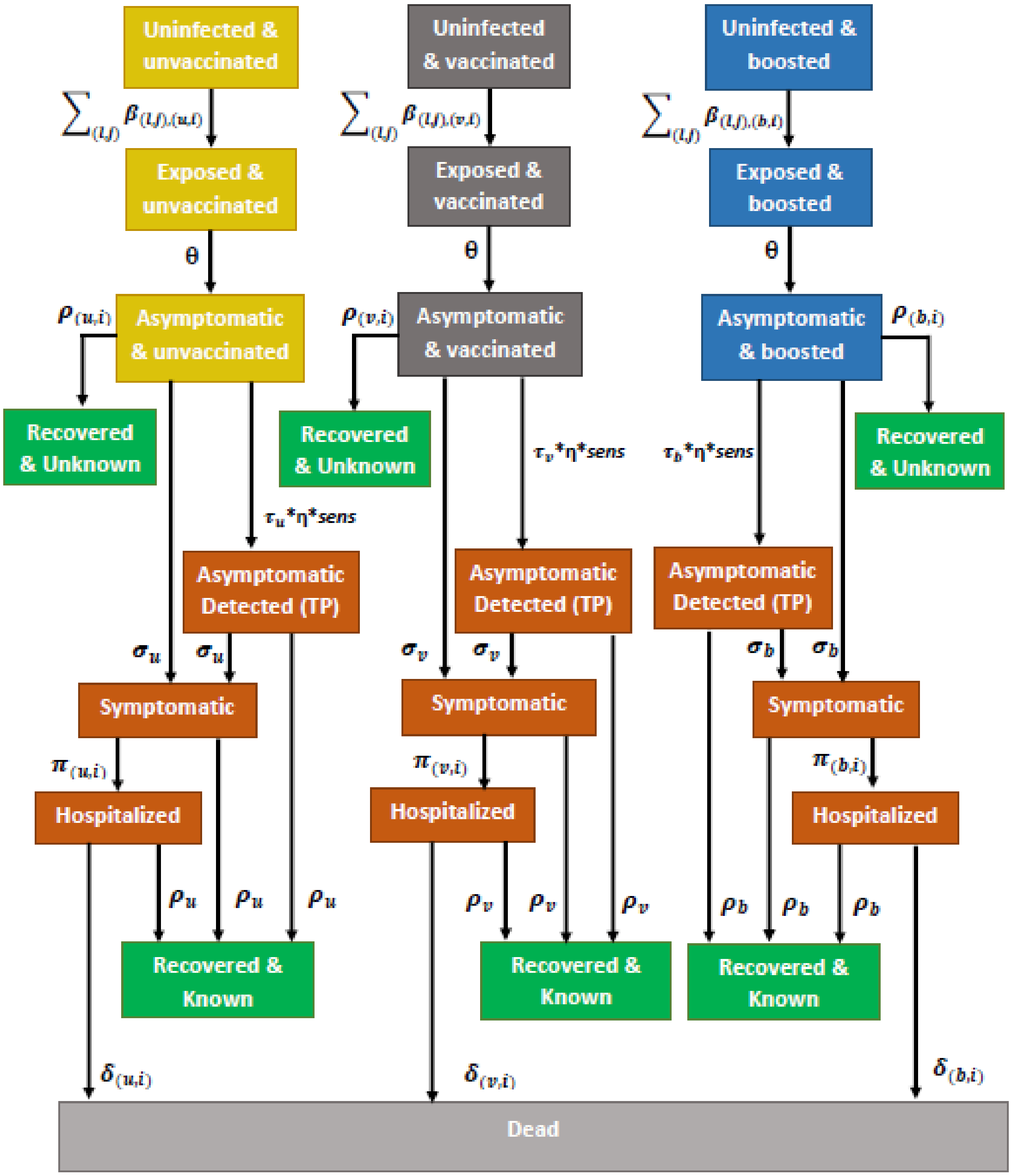

Supplementary Figure S1: Flow Diagram of the Extended SEIR Model

Compartments

Our model decomposes the population based on group-specific disease and transmission dynamics as well as vaccination status by creating compartments for each population group $i \in \{\text{(s)tudents, (f)aculty}\}$, and for each vaccination status $k \in \{\text{(u)nvaccinated, (v)accinated, and (b)oosted}\}$.

We also model that individuals develop (natural) immunity throughout the semester if they have recovered from an infection. Individuals who recover from an infection are assumed to be immune for the rest of the semester. In addition, we assume that the acquired natural immunity and the immunity of the individuals who are boosted do not wane throughout the semester since the it is not long enough (80 days). Accordingly, we have 62 compartments in total, which are categorized into *pools* and described in detail below.

Transmission pool contains those individuals who are either susceptible, or currently infected without symptoms and with an unknown infection status (i.e., without a positive test outcome), that is, it contains the following compartments for each group i & vaccination status k:

- $U_{k,i}$: Uninfected and susceptible.
- $E_{k,i}$: Exposed, asymptomatic, and non-infectious.
- $A_{k,i}$: Infected and asymptomatic.

Recovered pool contains those individuals who have recovered from a prior infection (unknowingly or unknowingly), and hence developed natural immunity, and contains the following compartments for each group i and vaccination status k:

- $RU_{k,i}$: Recovered & unknown.
- $RK_{k,i}$: Recovered & known.

Isolation/hospital pool contains those individuals who are in isolation or at the hospital. Accordingly, the individuals in this pool cannot transmit the infection, or if they are a false-positive, they cannot get infected. This pool contains the following compartments for each group i & vaccination status k:

- $FP_{k,i}$: False-positive with no prior infection (hence in isolation).
- $FPRU_{k,i}$: False-positive, and unknowingly recovered from a prior infection (hence in isolation).
- $TP_{k,i}$ (Asymptomatic Detected): True-positive, infected, and asymptomatic (hence in isolation).
- $S_{k,i}$: Infected and symptomatic (hence in isolation).
- $H_{k,i}$: Hospitalized.

Removed pool contains those individuals who have died, that is, for each group i, which are contained in the compartment, D_i, Dead.

Screening pool contains those individuals who are eligible for screening based on the given screening policy.

A summary of the characteristics of each compartment is given in Table S1.

Supplementary Table S1: Summary of Compartmental Characteristics

**Compartment**	**In screening pool?**	**In transmission pool?**	**In isolation/ hospital pool?**
($U_{u,i}$)–Uninfected & unvaccinated	Yes	Yes	No
($U_{v,i}$)–Uninfected & vaccinated	Depends on screening policy	Yes	No
($U_{b,i}$)–Uninfected & boosted	Depends on screening policy	Yes	No
($E_{u,i}$)–Exposed & unvaccinated	Yes	Yes	No
($E_{v,i}$)–Exposed & vaccinated	Depends on screening policy	Yes	No
($E_{b,i}$)–Exposed & boosted	Depends on screening policy	Yes	No
($A_{u,i}$)–Asymptomatic & unvaccinated	Yes	Yes	No
($A_{v,i}$)–Asymptomatic & vaccinated	Depends on screening policy	Yes	No
($A_{b,i}$)–Asymptomatic & boosted	Depends on screening policy	Yes	No
($RU_{k,i}$)–Recovered and unknown	Depends on screening policy	No	No
($RK_{k,i}$)–Recovered and known	No	No	No
($FP_{k,i}$)–False-positive with no prior infection	No	No	Yes
($FPRU_{k,i}$)–False-positive, unknowingly recovered from a prior infection	No	No	Yes
($TP_{k,i}$)–True-positive, Asymptomatic detected	No	No	Yes
($S_{k,i}$)–Symptomatic	No	No	Yes
($H_{k,i}$)–Hospitalized	No	No	Yes
(D_i)–Dead	No	No	No

Model Parameters

Subscript $i, j \in \{s, f\}$ denotes the the population group, and subscripts $k, l \in \{u, v, b\}$ denote the vaccination status. When a subscript is omitted, those parameter values apply to all values of the omitted subscript, that is, parameters without the group index i apply to both student and faculty groups, whereas those without any index apply to the entire population (i.e., both groups and all vaccination categories). When needed, we use the superscript"ω_O" to denote probabilistic conditioning, to model the setting where a certain fraction (ω_O) of all COVID-19 infections are caused by the Omicron variant, and the remainder is caused by the Delta variant. We also use the time index (t) to indicate that the corresponding parameter varies over time t according to the dynamics of the model.

Parameters related to infection prevalence and spread:

ω_O: percentage of all COVID-19 infections caused by the Omicron variant (with the remaining $1 - \omega_O\%$ caused by the Delta variant)

$R0^{\omega_O}_{(l,j)}$: basic reproduction number for subjects in group j & vaccination status l, for a given ω_O

$\beta^{\omega_O}_{(l,j),(k,i)}(t)$ rate at which infected subjects in group j & vaccination status l contact and infect subjects in group i & vaccination status k, for a given ω_O

$\gamma \in (0, 1)$: reduction in disease transmission rate if a face mask policy is implemented

$I_{(k,i)}(t)$: an indicator function, which takes a value of 1 if an exogenous shock takes place in cycle t for group i & vaccination status k; and 0 otherwise

$X_{(k,i)}$: number of imported infections per week for subjects in group i & vaccination status k if an exogenous shock takes place in that week

Parameters related to infection outcomes:

$\epsilon^{\omega_O}_k$: vaccine effectiveness against infection for subjects with vaccination status $k \in \{v, b\}$, calculated as a weighted average considering an Omicron percentage of ω_O

$\upsilon^{\omega_O}_k$: vaccine effectiveness against hospitalization for subjects with vaccination status $k \in \{v, b\}$, calculated as a weighted average considering an Omicron percentage of ω_O

θ: rate at which exposed subjects become asymptomatic and infectious

σ_k: rate of symptom onset for infected subjects in vaccination status k

$\delta^{\omega_O}_{(k,i)}$: fatality rate for subjects in group i & vaccination status k who are hospitalized

$\pi^{\omega_O}_{(k,i)}$: hospitalization rate for subjects in group i & vaccination status k who are symptomatic

ρ_k: recovery rate for infected subjects in vaccination status k

Parameters related to testing:

$sens$: sensitivity of the screening test

$spec$: specificity of the screening test

η: screening compliance rate

μ: rate at which subjects with false-positive outcomes return to the uninfected compartment

τ_k: screening rate for subjects with vaccination status k

The model uses a cycle time of 8 hours, that is, the number of subjects in each compartment is

updated every 8 hours. Screening rate for subjects in each vaccination status, $\tau_k, \forall k$, remains the same throughout the semester. Certain parameter values are varied in the analysis to simulate different strategies or scenarios, e.g., screening frequency, screening compliance rate, etc.

Governing Equations

The following defines the governing equations for the model depicted in Fig. S1, where $i, j \in \{s, f\}$ and $k, l \in \{u, v, b\}$. Let $Z_{k,i}(t) \equiv U_{k,i}(t) + E_{k,i}(t) + A_{k,i}(t) + RU_{k,i}(t) + RK_{k,i}(t)$.

$$U_{u,i}(t+1) = U_{u,i}(t) \times \left[1 - \sum_l \sum_j \left[\beta^{\omega_O}_{(l,j),(u,i)}(t) \times \frac{A_{l,j}(t)}{Z_{u,i}(t)}\right]\right] - U_{u,i}(t-1) \times \tau_u \times \eta \times (1 - spec)$$
$$+ \mu \times FP_{u,i}(t) - X_{(u,i)} \times I_{(u,i)}(t+1)$$

$$U_{v,i}(t+1) = U_{v,i}(t) \times \left[1 - \sum_l \sum_j \left[\beta^{\omega_O}_{(l,j),(v,i)}(t) \times \frac{A_{l,j}(t)}{Z_{v,i}(t)}\right]\right] - U_{v,i}(t-1) \times \tau_v \times \eta \times (1 - spec)$$
$$+ \mu \times FP_{v,i}(t) - X_{(v,i)} \times I_{(v,i)}(t+1)$$

$$U_{b,i}(t+1) = U_{b,i}(t) \times \left[1 - \sum_l \sum_j \left[\beta^{\omega_O}_{(l,j),(b,i)}(t) \times \frac{A_{l,j}(t)}{Z_{b,i}(t)}\right]\right] - U_{b,i}(t-1) \times \tau_b \times \eta \times (1 - spec)$$
$$+ \mu \times FP_{b,i}(t) - X_{(b,i)} \times I_{(b,i)}(t+1)$$

$$E_{k,i}(t+1) = E_{k,i}(t) \times [1 - \theta] + \sum_l \sum_j \left[\beta^{\omega_O}_{(l,j),(k,i)}(t) \times \frac{U_{k,i}(t) \times A_{l,j}(t)}{Z_{k,i}(t)}\right] + X_{(k,i)} \times I_{(k,i)}(t+1)$$

$$A_{k,i}(t+1) = A_{k,i}(t) \times [1 - \sigma_k - \rho_k] - A_{k,i}(t-1) \times \tau_k \times \eta \times sens + E_{k,i}(t) \times \theta$$

$$FP_{k,i}(t+1) = FP_{k,i}(t) \times [1 - \mu] + U_{k,i}(t-1) \times \tau_k \times \eta \times (1 - spec)$$

$$TP_{k,i}(t+1) = TP_{k,i}(t) \times [1 - \sigma_k - \rho_k] + A_{k,i}(t-1) \times \tau_k \times \eta \times sens$$

$$S_{k,i}(t+1) = S_{k,i}(t) \times [1 - \rho_k - \pi^{\omega_O}_{(k,i)}] + \sigma_k \times [TP_{k,i}(t) + A_{k,i}(t)]$$

$$H_{k,i}(t+1) = H_{k,i}(t) \times [1 - \rho_k - \delta^{\omega_O}_{(k,i)}] + \pi^{\omega_O}_{(k,i)} \times S_{k,i}(t)$$

$$RK_{k,i}(t+1) = RK_{k,i}(t) + \rho_k \times \left[TP_{k,i}(t) + S_{k,i}(t) + H_{k,i}(t)\right]$$

$$RU_{k,i}(t+1) = RU_{k,i}(t) + \rho_k \times A_{k,i}(t) - RU_{k,i}(t-1) \times \tau_k \times \eta \times (1 - spec) + \mu \times FPRU_{k,i}(t)$$

$$D_i(t+1) = D_i(t) + \sum_k \left[\delta^{\omega_O}_{(k,i)} \times H_{k,i}(t)\right]$$

$$FPRU_{k,i}(t+1) = FPRU_{k,i}(t) \times [1-\mu] + RU_{k,i}(t-1) \times \tau_k \times \eta \times (1 - spec)$$

$$N = \sum_k \sum_i \Big[U_{k,i} + E_{k,i} + A_{k,i} + RU_{k,i} + FPRU_{k,i} + S_{k,i} + TP_{k,i} + FP_{k,i} + H_{k,i} + RK_{k,i}\Big] + \sum_i D_i$$

Initial Conditions

We assume a 15:1 student to faculty ratio and a medium size college campus, with a total population of 24,000 (22,500 students and 1,500 faculty members). We assume that any subject with some immunity at the beginning of the academic semester has acquired it through vaccination, but model that an individual can acquire natural immunity through an infection during the semester. We consider the following initial conditions, with multiple values for a parameter representing the values considered in sensitivity analysis:

- $A_{k,i}(0) = \begin{cases} 45, & i = s \\ 3, & i = f \end{cases}$
- $U_{u,i}(0) = \begin{cases} \{4,000\ ;\ 7,000\ ;\ 10,000\} - A_{u,i}(0), & i = s \\ \{267\ ;\ 467\ ;\ 667\} - A_{u,i}(0), & i = f \end{cases}$
- $U_{v,i}(0) = \begin{cases} \{4,000\ ;\ 7,000\ ;\ 10,000\} - A_{v,i}(0), & i = s \\ \{267\ ;\ 467\ ;\ 667\} - A_{v,i}(0), & i = f \end{cases}$
- $U_{b,i}(0) = \begin{cases} 22,500 - U_{u,i}(0) - U_{v,i}(0) - A_{b,i}(0) - A_{v,i}(0) - A_{u,i}(0), & i = s \\ 1,500 - U_{u,i}(0) - U_{v,i}(0) - A_{b,i}(0) - A_{v,i}(0) - A_{u,i}(0), & i = f \end{cases}$

All other compartments are initially empty. Accordingly, $N = 22,500 + 1,500 = 24,000$. We note that for a given coverage in students, it is assumed that the coverage in faculty is

15 times less than the coverage in students. This is also assumed to be true within each vaccination status as well, i.e., whenever there is 4K unvaccinated students, it is assumed that there will be 4K/15=267 unvaccinated faculty. In this paper, we consider two options for the coverage in students: (a) 4K unvaccinated, 4K vaccinated, 14.455K boosted, and (b) 4K unvaccinated, 10K vaccinated, 8.455K boosted. This is equivalent to: (a) 82% coverage (64% boosted, 18% vaccinated) and 18% unvaccinated, and (b) 82% coverage (38% boosted, 44% vaccinated) and 18% unvaccinated. We use the latter representation in this paper and omit "18% unvaccinated for simplicity."

Further, in the base case, we use the following values for $X_{k,i}$, the number of imported infections per week on subjects in group i & vaccination status k if an exogenous shock takes place in that week:

- $X_{u,i} = \begin{cases} 15, & i = s \\ 1 (= 15/15), & i = f \end{cases}$
- $X_{v,i} = \begin{cases} 10, & i = s \\ 2/3 (= 10/15), & i = f \end{cases}$
- $X_{b,i} = \begin{cases} 5, & i = s \\ 1/3 (= 5/15), & i = f \end{cases}$

Estimation of Key Parameters

We model that the two variants (Delta and Omicron) may be circulating simultaneously, where the parameter ω_0 represents the percentage of all infections caused by the Omicron variant (with $1 - \omega_0$ representing the percentage caused by the Delta variant). Because the reported basic reproduction numbers (R) and vaccine effectiveness values (ϵ, υ) differ for the Omicron and Delta variants, we compute the basic reproduction number and vaccine effectiveness values as weighted averages of the respective values for each variant, as a function of ω_0. We consider a 3:1 ratio between the R values for the Omicron and Delta variants [4, 7],

Supplementary Table S2: Fatality[1] and Hospitalization Rate Computations for Faculty and Student Groups

	**Students**		**Faculty**	
	**Hospitalization rate ($H_{(k,s)}$)**	**Fatality rate ($F_{(k,s)}$)**	**Hospitalization rate ($H_{(k,f)}$)**	**Fatality rate ($F_{(k,f)}$)**
**For general values of ω_O**				
**Unvaccinated**	1.4% [3,5,9]	0.05% [9,11,12]	8.4% [3,5,9]	2% [9,11,12]
**Vaccinated**	$1.4\% \times (1 - \upsilon_v^{\omega_O})$	$0.05\% \times (1 - 1.002 \times \upsilon_v^{\omega_O})$	$8.4\% \times (1 - \upsilon_v^{\omega_O})$	$2\% \times (1 - 1.002 \times \upsilon_v^{\omega_O})$
**Boosted**	$1.4\% \times (1 - \upsilon_b^{\omega_O})$	$0.05\% \times (1 - 1.002 \times \upsilon_b^{\omega_O})$	$8.4\% \times (1 - \upsilon_b^{\omega_O})$	$2\% \times (1 - 1.002 \times \upsilon_b^{\omega_O})$
**When $\omega_O = 50\%$**				
**Unvaccinated**	1.4%	0.05%	8.4%	2%
**Vaccinated**	0.2681%	0.009494%	1.6086%	0.3798%
**Boosted**	0.0665%	0.00228%	0.399%	0.09119%
k	$\pi_{(k,s)}^{\omega_O}$	$\delta_{(k,s)}^{\omega_O}$	$\pi_{(k,f)}^{\omega_O}$	$\delta_{(k,f)}^{\omega_O}$
**Unvaccinated**	0.003261	0.002469	0.0259	0.02083
**Vaccinated**	0.0006007	0.002447	0.003774	0.0206
**Boosted**	0.000148	0.002367	0.000898	0.01975

[1] Vaccine effectiveness against fatality is assumed to be 0.2% higher than vaccine effectiveness against hospitalization

see Table 3 of the Manuscript. Then using these estimates, we find the parameter values reported in Table S2.

In the following, we provide the detailed calculations and references for the computed parameters:

- ω_O, the percentage of all COVID-19 infections caused by the Omicron variant (with the remaining $1-\omega_0$ caused by the Delta variant). Baseline value of $\omega_O = 75\%$; sensitivity analysis over $\omega_O = \{50\%, 75\%, 95\%\}$.

- $R0_{(l,j)}^{\omega_O}$, basic reproduction number for subjects in group j & vaccination status l, for a given ω_O. We assume that for $j \in \{s, f\}$, $R0_{(v,j)}^{\omega_O}$ and $R0_{(b,j)}^{\omega_O}$ are equal to $R0_{(u,j)}^{\omega_O}$ because, once infected, vaccinated subjects are thought to transmit COVID-19 similarly to unvaccinated subjects [10]. Then, we compute $R0_{(u,j)}^{\omega_O}$ as a weighted average of the respective values for the Delta and Omicron variants (see Table 3 of the Manuscript), assuming that Omicron 3 times as infectious as Delta [4, 7]. As of December, 2021, the basic reproduction number for the Delta variant is reported to be between 2 and 8 [6]; and we assume that the basic reproduction number of Delta is 3.2 for the faculty and 6 for the students in the base-case transmission scenario and 2.2/5 and 4.2/7 for the faculty/students in the best- and worst-case scenarios, respectively. Then,

when $\omega_O = 50\%$, $R0_{(l,s)}^{\omega_O=50\%} = 12$ and $R0_{(l,f)}^{\omega_O=50\%} = 6.4$, $l \in \{u, v, b\}$ (Table 3 of the Manuscript).

- All vaccine effectiveness values ($\epsilon_k^{\omega_O}, \upsilon_k^{\omega_O}, k \in \{v, b\}$) are computed as weighted averages of the respective values for the Delta and Omicron variants, for a given ω_O (see Table 3 of the Manuscript). For example, these calculations yield the following numbers for the case of $\omega_0 = 50\%$: $\epsilon_v^{\omega_O=50\%} = 56.5\%$ and $\epsilon_b^{\omega_O=50\%} = 78.05\%$ (i.e., vaccine effectiveness against infection in vaccinated and boosted individuals, respectively), and $\upsilon_v^{\omega_O=50\%} = 80.85\%$ and $\upsilon_b^{\omega_O=50\%} = 95.25\%$ (i.e., vaccine effectiveness against hospitalization in vaccinated and boosted individuals, respectively), see Table 3 of the Manuscript.

- $\beta_{(l,j),(k,i)}^{\omega_O}(t)$, rate at which infected subjects in group j & vaccination status l contact and infect subjects in group i & vaccination status k, for a given ω_O. This rate depends on transmission severity, represented in terms of the reproduction number $R_{(l,j),(k,i)}^{\omega_O}$. We model β as a function of time (t) due to the change in the fraction of susceptibles over time, thus extending the concept of the time-varying reproduction number, described in [1], to a population of subjects with different vaccination status. In particular, in time period t, the total number of susceptibles, $N_{su}(t) \equiv \sum_i \sum_k U_{k,i}(t)$, hence we can write, for $i, j \in \{s, f\}, l \in \{u, v, b\}$:
$$\begin{aligned} R_{(l,j),(u,i)}^{\omega_O}(t+1) &= \left[(1-\gamma) \times R0_{(l,j)}^{\omega_O} \times \frac{U_{u,i}(t)}{N_{su}(t)}\right] \\ R_{(l,j),(v,i)}^{\omega_O}(t+1) &= \left[(1-\gamma) \times R0_{(l,j)}^{\omega_O} \times \frac{U_{v,i}(t)}{N_{su}(t)} \times (1-\epsilon_v^{\omega_O})\right] \\ R_{(l,j),(b,i)}^{\omega_O}(t+1) &= \left[(1-\gamma) \times R0_{(l,j)}^{\omega_O} \times \frac{U_{b,i}(t)}{N_{su}(t)} \times (1-\epsilon_b^{\omega_O})\right]. \end{aligned}$$
Then, $\beta_{(l,j),(k,i)}^{\omega_O}(t)$ is the solution to:
$$R_{(l,j),(k,i)}^{\omega_O}(t) = \beta_{(l,j),(k,i)}^{\omega_O}(t)/(\sigma_k + \rho_k) \Rightarrow \beta_{(l,j),(k,i)}^{\omega_O}(t) = R_{(l,j),(k,i)}^{\omega_O}(t) \times (\sigma_k + \rho_k).$$

- $\pi_{(k,i)}^{\omega_O}$, hospitalization rate for subjects in group i & vaccination status k who are symptomatic. These rates are calculated based on hospitalization rates, denoted by H (Table S2), and vaccine effectiveness against hospitalization in vaccinated and boosted sub-

jects $((\upsilon_v^{\omega_O}), (\upsilon_b^{\omega_O})$, Table 3 of the Manuscript). Then, for $i \in \{s, f\}$ and $k \in \{u, v, b\}$, $\pi_{(k,i)}^{\omega_O}$ is the solution to:

$[\sigma_k/(\rho_k + \sigma_k)] \times [\pi_{(k,i)}^{\omega_O}/(\rho_k + \pi_{(k,i)}^{\omega_O})] = H_{(k,i)} \Rightarrow \pi_{(k,i)}^{\omega_O} = \rho_k \times H_{(k,i)}/\big([\sigma_k/(\rho_k + \sigma_k)] - H_{(k,i)}\big)$;

see Table S2 for values of $\pi_{(k,i)}^{\omega_O=50\%}, i \in \{s, f\}, k \in \{u, v, b\}$.

- $\delta_{(k,i)}^{\omega_O}$, fatality rate for subjects in group i & vaccination status k who are hospitalized. These rates are calculated based on fatality rates, denoted by F (Table S2), and vaccine effectiveness against death in vaccinated and boosted subjects, assumed to be 0.2% higher than vaccine effectiveness against hospitalization (i.e., $1.002 \times \epsilon_v^{\omega_O}$ and $1.002 \times \epsilon_b^{\omega_O}$, respectively, see Table 3 of the Manuscript). Then, for $i \in \{s, f\}$ and $k \in \{u, v, b\}$, $\delta_{(k,i)}^{\omega_O}$ is the solution to:

$$[\sigma_k/(\rho_k + \sigma_k)] \times [\delta_{(k,i)}^{\omega_O}/(\rho_k + \delta_{(k,i)}^{\omega_O})] \times [\pi_{(k,i)}/(\rho_k + \pi_{(k,i)})] = F_{(k,i)}$$

$$\Rightarrow \delta_{(k,i)}^{\omega_O} = \rho_k \times F_{(k,i)}/\big([\sigma_k/(\rho_k + \sigma_k)] \times [\pi_{(k,i)}^{\omega_O}/(\rho_k + \pi_{(k,i)}^{\omega_O})] - F_{(k,i)}\big).$$

see Table S2 for values of $\delta_{(k,i)}^{\omega_O=50\%}, i \in \{s, f\}, k \in \{u, v, b\}$.

The following include the parameters that are independent of ω_O:

- θ, rate at which exposed subjects become Asymptomatic & infectious: It is given by $\theta = \frac{1}{33.5} = 0.095$ since the mean latent period is 3.5 days and each day is composed of 3 eight-hour cycles.

- σ_k, rate of symptom onset in infected individuals with vaccination status k: σ_k is the solution to, $\sigma_k/(\sigma_k + \rho_k) = 30\%$, where 30% is the probability of developing symptoms after exposure [8], which, in the absence of reliable data, is assumed to be the same for all subjects; and ρ_k is the recovery rate of infected individuals in vaccination status k, which, again in the absence of reliable data, is assumed to be the same for all subjects, and is derived from the time to recovery, assumed to be 5 days for all infected individuals [2] (independently of vaccination status and age), where each day is composed of three 8-hour cycles. Then, $\rho_k = 1/(3 \times 5)$, leading to $\sigma_k = 0.0286,\ k \in \{u, v, b\}$.

- τ_k, screening rate for subjects with vaccination status k: We have, $\tau_k = 1/(3 \times f_k)$, where f_k is the screening frequency for vaccination status k, which can be *daily, every 2 days, every 3 days, every 7 days, or every 14 days.*

- γ, reduction in disease transmission rate if a face mask policy is implemented. We assume it to be $\gamma = 0.5$ based on [13].

- η, screening compliance rate: Baseline value of $\eta = 0.75$; with sensitivity analysis over $\eta = \{0.75, 0.90\}$.

Fig. S1 presents a flow diagram of the extended SEIR model. To improve the clarity, Fig. S1 does not include the false-positive compartments, and for those compartments that are defined for both student and faculty groups, only one compartment is shown in the figure. Next, we enclose some results in the following tables. We note that $S/\overline{S}$ denotes screening/no screening, and $u/v/b$ denotes unvaccinated/vaccinated/boosted vaccination status. Thus, S_u and $S_{u,v}$ represent strategies that customize the screening population, $S_{u,v,b}$ represents universal screening, and $\overline{S}$ represents no screening.

Supplementary Table S3: Parameter Values and Sensitivity Analysis

**Model Parameter**	**Value(s)**	**Input for:**
**Disease related**		
Proportion of infections<br>due to the Omicron variant (ω_0)	50%, 95%	$R0^{\omega_O}_{(k,i)}$, $\epsilon^{\omega_O}_m$, $\upsilon^{\omega_O}_m$, $H_{(m,i)}$, $F_{(m,i)}$,<br>$R^{\omega_O}_{(l,j),(k,i)}(t)$, $\beta^{\omega_O}_{(l,j),(k,i)}(t)$,<br>$\pi^{\omega_O}_{(m,i)}$, $\delta^{\omega_O}_{(m,i)}$
Mean incubation time	3.5 days[1]	θ
Time to recovery	5 days	σ_k, $\delta^{\omega_O}_{(k,i)}$, $\pi^{\omega_O}_{(k,i)}$
**Infectiousness**		
Infectiousness (basic reproduction number) ratio:<br>Omicron:Delta	3:1	$R0^{\omega_O}_{(k,s)}$, $R^{\omega_O}_{(l,s),(k,i)}(t)$, $\beta^{\omega_O}_{(l,s),(k,i)}(t)$<br>$R0^{\omega_O}_{(k,f)}$, $R^{\omega_O}_{(l,f),(k,i)}(t)$, $\beta^{\omega_O}_{(l,f),(k,i)}(t)$
**Inputs for basic reproduction number of (variant, group):**		
Delta, students	5, 6, 7	$R0^{\omega_O}_{(k,s)}$, $R^{\omega_O}_{(l,s),(k,i)}(t)$, $\beta^{\omega_O}_{(l,s),(k,i)}(t)$
Omicron, students	$3 \times \{5, 6, 7\}$	$R0^{\omega_O}_{(k,s)}$, $R^{\omega_O}_{(l,s),(k,i)}(t)$, $\beta^{\omega_O}_{(l,s),(k,i)}(t)$
Delta, faculty	2.2, 3.2, 4.2	$R0^{\omega_O}_{(k,f)}$, $R^{\omega_O}_{(l,f),(k,i)}(t)$, $\beta^{\omega_O}_{(l,f),(k,i)}(t)$
Omicron, faculty	$3 \times \{2.2, 3.2, 4.2\}$	$R0^{\omega_O}_{(k,f)}$, $R^{\omega_O}_{(l,f),(k,i)}(t)$, $\beta^{\omega_O}_{(l,f),(k,i)}(t)$
Reduction in disease transmission rate<br>under a face mask policy (γ)	50%	$R^{\omega_O}_{(l,j),(k,i)}(t)$, $\beta^{\omega_O}_{(l,j),(k,i)}(t)$
**Disease outcomes**		
**Vaccine effectiveness against infection for (variant, vaccination status):**		
Delta, vaccinated	80%	$\epsilon^{\omega_O}_v$, $R^{\omega_O}_{(l,j),(v,i)}(t)$, $\beta^{\omega_O}_{(l,j),(v,i)}(t)$
Omicron, vaccinated	33%	$\epsilon^{\omega_O}_v$, $R^{\omega_O}_{(l,j),(v,i)}(t)$, $\beta^{\omega_O}_{(l,j),(v,i)}(t)$
Delta, boosted	86.7%	$\epsilon^{\omega_O}_b$, $R^{\omega_O}_{(l,j),(b,i)}(t)$, $\beta^{\omega_O}_{(l,j),(b,i)}(t)$
Omicron, boosted	69.4%	$\epsilon^{\omega_O}_b$, $R^{\omega_O}_{(l,j),(b,i)}(t)$, $\beta^{\omega_O}_{(l,j),(b,i)}(t)$
Symptom development rate for infected<br>(all vaccination status)	30%	σ_k, $\delta^{\omega_O}_{(k,i)}$, $\pi^{\omega_O}_{(k,i)}$
Hospitalization rate for symptomatic<br>for unvaccinated (students/faculty)	1.4% / 8.4%	$H_{(k,i)}$, $\pi^{\omega_O}_{(k,i)}$
**Vaccine effectiveness against hospitalization for symptomatic (variant, vaccination status):**		
Omicron, vaccinated	70%	$\upsilon^{\omega_O}_v$, $H_{(v,i)}$, $F_{(v,i)}$, $\pi^{\omega_O}_{(v,i)}$, $\delta^{\omega_O}_{(v,i)}$
Delta, vaccinated	91.7%	$\upsilon^{\omega_O}_v$, $H_{(v,i)}$, $F_{(v,i)}$, $\pi^{\omega_O}_{(v,i)}$, $\delta^{\omega_O}_{(v,i)}$
Omicron, boosted	93%	$\upsilon^{\omega_O}_b$, $H_{(b,i)}$, $F_{(b,i)}$, $\pi^{\omega_O}_{(b,i)}$, $\delta^{\omega_O}_{(b,i)}$
Delta, boosted	97.5%	$\upsilon^{\omega_O}_b$, $H_{(b,i)}$, $F_{(b,i)}$, $\pi^{\omega_O}_{(b,i)}$, $\delta^{\omega_O}_{(b,i)}$
Fatality rate for hospitalized<br>for unvaccinated (students/faculty)	0.05% / 2%	$F_{(k,i)}$, $\delta^{\omega_O}_{(k,i)}$
**Screening test characteristics**		
Test sensitivity (*sens*)	80%	
Test specifity (*spec*)	98%	

[1] Average of the 3- and 4-day mean incubation times for Omicron and Delta, respectively

Supplementary Table S4: Number of infections (unvaccinated, vaccinated, boosted, total), number of deaths, number of hospitalizations (unvaccinated, vaccinated, boosted, total) over the 80-day semester for various screening strategies, considering that 50% of the infections are caused by Omicron ($\omega_0 = 50\%$), 75% screening compliance ($\eta = 75\%$), and various coverage.

		Tests	Infections					Deaths	Hospitalizations				
Strategy	Screening frequency	Average per day	Peak (daily)	Unvaccinated	Vaccinated	Boosted	Total	Total	Peak (daily)	Unvaccinated	Vaccinated	Boosted	Total
**82% coverage (64% boosted, 18% vaccinated)**													
$\overline{S}$	N/A	0	180	3,729	3,356	10,522	17,606	9	12	72	12	9	93
S_u	every 14d	179	167	3,692	3,287	10,176	17,155	8	11	71	12	9	92
	every 7d	342	156	3,658	3,225	9,876	16,759	8	11	70	12	8	90
	every 3 d	739	133	3,577	3,088	9,229	15,893	8	10	69	11	8	87
	every 2d	1,070	120	3,516	2,989	8,781	15,286	8	9	67	11	7	85
	every 1d	2,065	96	3,366	2,762	7,811	13,940	7	7	63	10	6	79
$S_{u,v}$	every 14d	370	159	3,656	3,227	9,859	16,741	8	11	70	12	8	90
	every 7d	720	141	3,580	3,099	9,240	15,919	8	10	69	11	8	88
	every 3 d	1,635	103	3,365	2,757	7,737	13,859	7	8	64	10	6	80
	every 2d	2,468	81	3,155	2,455	6,558	12,168	7	7	59	8	5	73
	every 1d	5,202	45	2,458	1,634	3,875	7,967	5	4	44	5	3	53
$S_{u,v,b}$	every 14d	1,113	142	3,538	3,023	8,897	15,458	8	10	68	11	8	87
	every 7d	2,233	109	3,293	2,640	7,260	13,193	7	8	63	9	6	79
	every 3d	5,429	48	2,376	1,546	3,585	7,507	5	5	45	6	3	54
	every 2d	8,405	31	1,497	826	1,735	4,058	3	2	29	3	1	34
	every 1d	17,265	31	427	199	386	1,012	1	1	11	1	0	12
**82% coverage (38% boosted, 44% vaccinated)**													
$\overline{S}$	N/A	0	263	3,873	9,210	6,959	20,042	11	16	75	33	6	114
S_u	every 14d	173	249	3,852	9,102	6,818	19,772	10	16	74	32	6	113
	every 7d	328	238	3,833	9,010	6,699	19,541	10	15	74	32	6	112
	every 3 d	691	214	3,792	8,817	6,456	19,065	10	14	73	31	6	110
	every 2d	980	200	3,763	8,691	6,301	18,755	10	14	73	31	5	109
	every 1d	1,807	174	3,701	8,440	6,002	18,142	10	12	71	30	5	106
$S_{u,v}$	every 14d	637	229	3,795	8,838	6,464	19,096	10	15	73	31	6	110
	every 7d	1,238	199	3,708	8,442	5,975	18,125	10	14	72	30	5	107
	every 3 d	2,854	135	3,429	7,278	4,715	15,422	9	10	66	25	4	95
	every 2d	4,402	94	3,116	6,135	3,681	12,932	8	8	59	21	3	83
	every 1d	9,703	36	1,920	2,944	1,477	6,341	4	3	36	9	1	46
$S_{u,v,b}$	every 14d	1,057	219	3,747	8,609	6,191	18,546	10	14	72	31	5	108
	every 7d	2,095	178	3,588	7,901	5,376	16,865	9	13	69	28	5	102
	every 3d	5,080	93	2,964	5,609	3,255	11,829	7	8	57	19	3	79
	every 2d	8,028	47	2,181	3,503	1,794	7,478	5	5	41	12	1	55
	every 1d	17,111	36	573	701	311	1,584	1	1	13	3	0	16

Supplementary Table S5: Number of infections (unvaccinated, vaccinated, boosted, total), number of deaths, number of hospitalizations (unvaccinated, vaccinated, boosted, total) over the 80-day semester for various screening strategies, considering that 95% of the infections are caused by Omicron ($\omega_0 = 95\%$), 75% screening compliance ($\eta = 75\%$), and various coverage.

		Tests	Infections					Deaths	Hospitalizations				
Strategy	Screening frequency	Average per day	Peak (daily)	Unvaccinated	Vaccinated	Boosted	Total	Total	Peak (daily)	Unvaccinated	Vaccinated	Boosted	Total
**82% coverage (64% boosted, 18% vaccinated)**													
$\overline{S}$	N/A	0	512	4,097	4,091	14,782	22,970	11	22	78	23	19	119
	every 14d	162	498	4,091	4,083	14,722	22,896	11	22	78	22	18	119
	every 7d	300	486	4,087	4,076	14,673	22,835	11	22	78	22	18	119
S_u	every 3 d	597	459	4,076	4,060	14,568	22,705	11	21	78	22	18	119
	every 2d	812	442	4,069	4,051	14,504	22,624	11	21	78	22	18	119
	every 1d	1,361	409	4,055	4,032	14,382	22,468	11	20	78	22	18	118
	every 14d	327	487	4,085	4,075	14,659	22,820	11	22	78	22	18	119
	every 7d	608	465	4,074	4,060	14,545	22,680	11	21	78	22	18	119
$S_{u,v}$	every 3 d	1,236	414	4,047	4,024	14,279	22,351	11	20	78	22	18	118
	every 2d	1,718	380	4,027	3,997	14,088	22,111	11	19	78	22	18	117
	every 1d	3,091	312	3,977	3,932	13,669	21,578	11	17	77	22	17	115
	every 14d	949	465	4,063	4,041	14,408	22,512	11	21	78	22	18	118
	every 7d	1,805	419	4,019	3,976	13,936	21,931	11	20	78	22	17	117
$S_{u,v,b}$	every 3d	4,017	306	3,843	3,722	12,228	19,794	10	17	74	21	15	110
	every 2d	6,206	222	3,608	3,396	10,323	17,327	9	14	70	19	13	102
	every 1d	15,073	61	2,334	1,902	4,331	8,568	6	6	45	10	5	61
**82% coverage (38% boosted, 44% vaccinated)**													
$\overline{S}$	N/A	0	658	4,133	10,482	8,828	23,443	14	30	78	56	11	146
	every 14d	160	645	4,130	10,471	8,808	23,409	14	30	78	56	11	146
	every 7d	294	635	4,128	10,462	8,791	23,380	14	30	78	56	11	146
S_u	every 3 d	578	611	4,122	10,442	8,757	23,321	14	29	78	56	11	146
	every 2d	779	595	4,118	10,429	8,734	23,281	14	29	78	56	11	146
	every 1d	1,270	563	4,110	10,405	8,692	23,206	14	28	78	56	11	145
	every 14d	565	623	4,121	10,442	8,751	23,314	14	29	78	56	11	146
	every 7d	1,049	590	4,108	10,397	8,665	23,170	14	29	78	56	11	145
$S_{u,v}$	every 3 d	2,128	509	4,069	10,264	8,420	22,753	13	27	78	55	11	144
	every 2d	2,971	448	4,031	10,137	8,197	22,366	13	25	78	55	10	143
	every 1d	5,585	314	3,911	9,736	7,549	21,197	13	21	76	52	9	137
	every 14d	919	612	4,113	10,411	8,697	23,221	14	29	78	56	11	145
	every 7d	1,722	566	4,087	10,314	8,525	22,925	13	28	78	56	11	145
$S_{u,v,b}$	every 3d	3,658	450	3,978	9,915	7,848	21,741	13	25	77	53	10	140
	every 2d	5,438	357	3,830	9,384	7,026	20,241	12	22	74	51	9	133
	every 1d	12,919	143	3,023	6,766	4,014	13,803	9	12	59	36	5	100

Supplementary Table S6: Number of infections (unvaccinated, vaccinated, boosted, total), number of deaths, number of hospitalizations (unvaccinated, vaccinated, boosted, total) over the 80-day semester for various screening strategies, considering that 95% of the infections are caused by Omicron ($\omega_0 = 95\%$), 82% coverage (with 64% boosted), and various screening compliance rates.

		Tests	Infections					Deaths	Hospitalizations				
Strategy	Screening frequency	Average per day	Peak (daily)	Unvaccinated	Vaccinated	Boosted	Total	Total	Peak (daily)	Unvaccinated	Vaccinated	Boosted	Total
**Screening compliance** $\eta = 75\%$													
$\overline{S}$	N/A	0	512	4,097	4,091	14,782	22,970	11	22	78	23	19	119
S_u	every 14d	162	498	4,091	4,083	14,722	22,896	11	22	78	22	18	119
	every 7d	300	486	4,087	4,076	14,673	22,835	11	22	78	22	18	119
	every 3 d	597	459	4,076	4,060	14,568	22,705	11	21	78	22	18	119
	every 2d	812	442	4,069	4,051	14,504	22,624	11	21	78	22	18	119
	every 1d	1,361	409	4,055	4,032	14,382	22,468	11	20	78	22	18	118
$S_{u,v}$	every 14d	327	487	4,085	4,075	14,659	22,820	11	22	78	22	18	119
	every 7d	608	465	4,074	4,060	14,545	22,680	11	21	78	22	18	119
	every 3 d	1,236	414	4,047	4,024	14,279	22,351	11	20	78	22	18	118
	every 2d	1,718	380	4,027	3,997	14,088	22,111	11	19	78	22	18	117
	every 1d	3,091	312	3,977	3,932	13,669	21,578	11	17	77	22	17	115
$S_{u,v,b}$	every 14d	949	465	4,063	4,041	14,408	22,512	11	21	78	22	18	118
	every 7d	1,805	419	4,019	3,976	13,936	21,931	11	20	78	22	17	117
	every 3d	4,017	306	3,843	3,722	12,228	19,794	10	17	74	21	15	110
	every 2d	6,206	222	3,608	3,396	10,323	17,327	9	14	70	19	13	102
	every 1d	15,073	61	2,334	1,902	4,331	8,568	6	6	45	10	5	61
**Screening compliance** $\eta = 90\%$													
$\overline{S}$	N/A	0	512	4,097	4,091	14,782	22,970	11	22	78	23	19	119
S_u	every 14d	191	495	4,090	4,081	14,713	22,885	11	22	78	22	18	119
	every 7d	350	481	4,085	4,073	14,654	22,812	11	22	78	22	18	119
	every 3 d	687	452	4,073	4,056	14,541	22,671	11	21	78	22	18	119
	every 2d	931	433	4,066	4,046	14,472	22,584	11	21	78	22	18	119
	every 1d	1,562	400	4,050	4,027	14,350	22,427	11	20	78	22	18	118
$S_{u,v}$	every 14d	386	482	4,083	4,072	14,635	22,791	11	22	78	22	18	119
	every 7d	711	456	4,070	4,055	14,501	22,626	11	21	78	22	18	119
	every 3 d	1,433	400	4,039	4,013	14,199	22,251	11	20	78	22	18	118
	every 2d	1,996	363	4,015	3,982	13,987	21,984	11	19	77	22	18	117
	every 1d	3,649	295	3,961	3,911	13,546	21,418	11	17	76	22	17	115
$S_{u,v,b}$	every 14d	1,125	455	4,055	4,029	14,323	22,407	11	21	78	22	18	118
	every 7d	2,134	401	3,998	3,946	13,720	21,663	11	20	77	22	17	116
	every 3d	4,850	270	3,759	3,603	11,502	18,865	10	16	73	20	14	107
	every 2d	7,714	178	3,424	3,152	9,079	15,655	9	12	66	17	11	95
	every 1d	19,090	56	1,670	1,272	2,621	5,563	4	3	32	7	3	43

Supplementary Table S7: Total number of infections and total number of hospitalizations over the 80-day semester for various screening strategies, considering various values for the percentage of infections caused by Omicron ($\omega_0 = 0\%, 50\%, 95\%$), 100% screening compliance ($\eta = 100\%$), and extreme cases of coverage.

		$\omega_O = 0\%$		$\omega_O = 50\%$		$\omega_O = 95\%$	
Strategy	Screening frequency	Total number of infections	Total number of hospitalizations	Total number of infections	Total number of hospitalizations	Total number of infections	Total number of hospitalizations
**Coverage: 100% boosted**							
	N/A	62	0	3,823	2	21,703	27
	every 14d	47	0	1,290	1	20,095	25
S_b	every 7d	38	0	569	1	17,913	21
	every 3 d	26	0	183	0	7,608	7
	every 2d	20	0	112	0	1,582	2
	every 1d	12	0	52	0	187	0
**Coverage: 100% vaccinated**							
	N/A	193	0	21,748	77	23,857	127
	every 14d	137	0	20,191	71	23,770	127
S_v	every 7d	106	0	18,094	62	23,619	126
	every 3 d	66	0	8,504	24	22,803	122
	every 2d	50	0	2,137	7	21,496	115
	every 1d	29	0	287	1	13,755	70
**Coverage: 0% (i.e., 100% unvaccinated)**							
	N/A	22,576	418	23,859	441	23,919	441
	every 14d	21,545	398	23,799	441	23,901	441
S_u	every 7d	20,160	371	23,689	439	23,889	441
	every 3 d	14,321	244	23,059	427	23,785	441
	every 2d	6,384	97	22,008	408	23,547	436
	every 1d	575	13	15,593	285	21,508	399

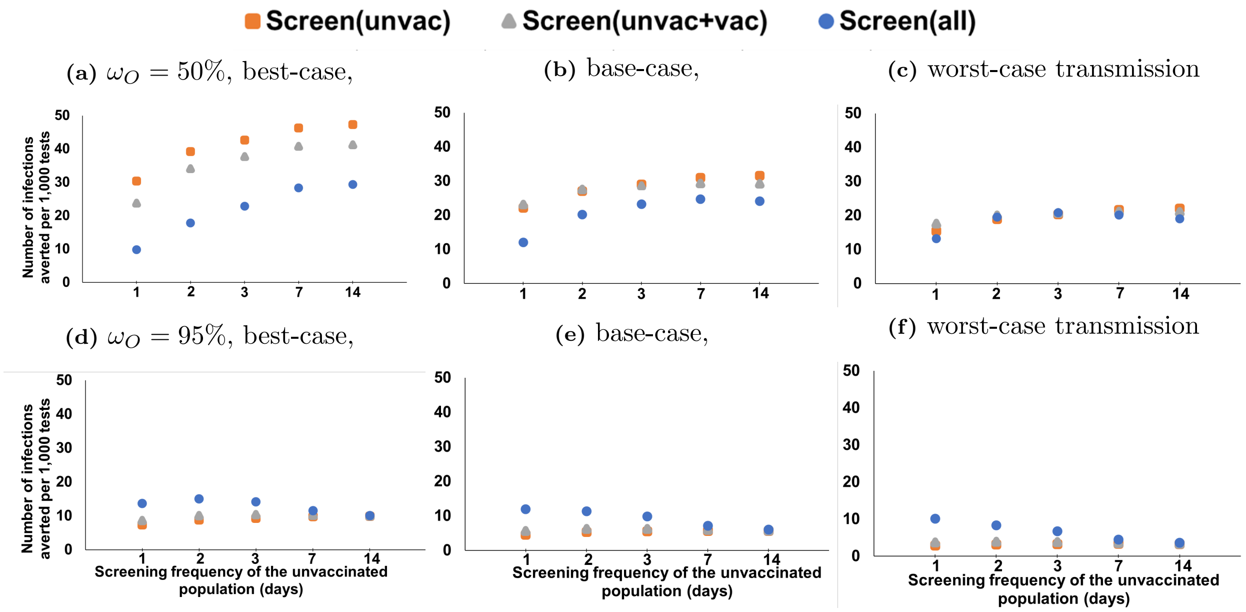

Supplementary Figure S2: Number of infections averted per 1,000 tests with respect to the screening frequency of the unvaccinated, for various Omicron proportions and transmission severity scenarios when the vaccination coverage is 64% boosted, 18% vac, 18% unvac (unvac: unvaccinated, vac: vaccinated)

Supplementary Table S8: Total number of infections and total number of hospitalizations over the 80-day semester for various screening strategies, considering various values for the percentage of infections caused by Omicron ($\omega_0 = 0\%, 50\%, 95\%$), 100% screening compliance ($\eta = 100\%$), high vaccine effectiveness against infection (as the Delta variant) and extreme cases of coverage.

		$\omega_O = 0\%$		$\omega_O = 50\%$		$\omega_O = 95\%$	
Strategy	Test Frequency	Total number of infections	Total number of hospitalizations	Total number of infections	Total number of hospitalizations	Total number of infections	Total number of hospitalizations
**Coverage: 100% boosted**							
	N/A	62	0	275	0	1,595	2
	every 14d	47	0	167	0	615	1
S_b	every 7d	38	0	119	0	327	0
	every 3 d	26	0	67	0	134	0
	every 2d	20	0	48	0	88	0
	every 1d	12	0	26	0	43	0
**Coverage: 100% vaccinated**							
	N/A	193	0	2,477	7	15,219	69
	every 14d	137	0	1,001	3	9,068	37
S_v	every 7d	106	0	536	2	4,079	17
	every 3 d	66	0	218	1	690	4
	every 2d	50	0	142	1	327	2
	every 1d	29	0	69	1	123	1
**Coverage: 0% (i.e., 100% unvaccinated)**							
	N/A	22,576	418	23,859	441	23,919	441
	every 14d	21,545	398	23,799	441	23,901	441
S_u	every 7d	20,160	371	23,689	439	23,889	441
	every 3 d	14,321	244	23,059	427	23,785	441
	every 2d	6,384	97	22,008	408	23,547	436
	every 1d	575	13	15,593	285	21,508	399

Supplementary Table S9: Number of infections averted per 1,000 tests with respect to the screening frequency of the unvaccinated population under different screening strategies and Omicron proportions for coverage of 82% (64% boosted, 18% vaccinated). (N/A indicates no screening)

Screening frequency of:				
Unvaccinated population	Vaccinated population	Boosted population	Screening strategy, label	Number of infections averted per 1,000 tests
$\omega_0 = 50\%$				
every 1d	N/A	N/A	customized screening population, S_u	22.2
	every 1d	N/A	customized screening population, $S_{u,v}$	23.2
	every 1d	every 1d	universal screening, $S_{u,v,b}$	12.0
	every 3d	N/A	customized screening population and frequency	27.8
every 2d	N/A	N/A	customized screening population, S_u	27.1
	every 2d	N/A	customized screening population, $S_{u,v}$	27.5
	every 2d	every 2d	universal screening, $S_{u,v,b}$	20.1
	every 7d	N/A	customized screening population and frequency	29.4
every 3d	N/A	N/A	customized screening population, S_u	29.0
	every 3d	N/A	customized screening population, $S_{u,v}$	28.7
	every 3d	every 3d	universal screening, $S_{u,v,b}$	23.3
	every 7d	N/A	customized screening population and frequency	30.2
every 7d	N/A	N/A	customized screening population, S_u	31.0
	every 7d	N/A	customized screening population, $S_{u,v}$	29.3
	every 7d	every 7d	universal screening, $S_{u,v,b}$	24.7
every 14d	N/A	N/A	customized screening population, S_u	31.6
	every 14d	N/A	customized screening population, $S_{u,v}$	29.2
	every 14d	every 14d	universal screening, $S_{u,v,b}$	24.1
$\omega_0 = 95\%$				
every 1d	N/A	N/A	customized screening population, S_u	4.6
	every 1d	N/A	customized screening population, $S_{u,v}$	5.6
	every 1d	every 1d	universal screening, $S_{u,v,b}$	11.9
	every 1d	every 2d	customized screening population and frequency	12.4
every 2d	N/A	N/A	customized screening population, S_u	5.3
	every 2d	N/A	customized screening population, $S_{u,v}$	6.3
	every 2d	every 2d	universal screening, $S_{u,v,b}$	11.4
every 3d	N/A	N/A	customized screening population, S_u	5.5
	every 3d	N/A	customized screening population, $S_{u,v}$	6.3
	every 3d	every 3d	universal screening, $S_{u,v,b}$	9.9
every 7d	N/A	N/A	customized screening population, S_u	5.6
	every 7d	N/A	customized screening population, $S_{u,v}$	6.0
	every 7d	every 7d	universal screening, $S_{u,v,b}$	7.2
every 14d	N/A	N/A	customized screening population, S_u	5.7
	every 14d	N/A	customized screening population, $S_{u,v}$	5.8
	every 14d	every 14d	universal screening, $S_{u,v,b}$	6.0

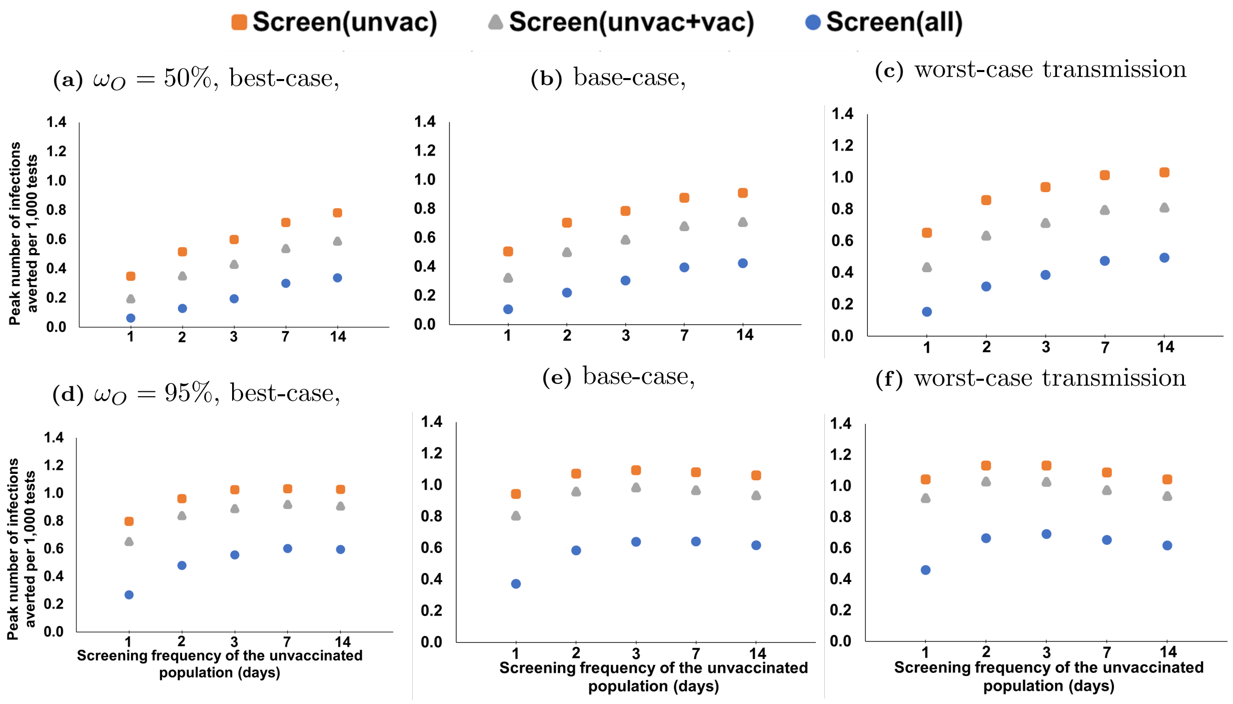

Supplementary Figure S3: Peak number of infections averted per 1,000 tests with respect to the screening frequency of the unvaccinated, for various Omicron proportions and transmission severity scenarios when the vaccination coverage is 64% boosted, 18% vac, 18% unvac (unvac: unvaccinated, vac: vaccinated)

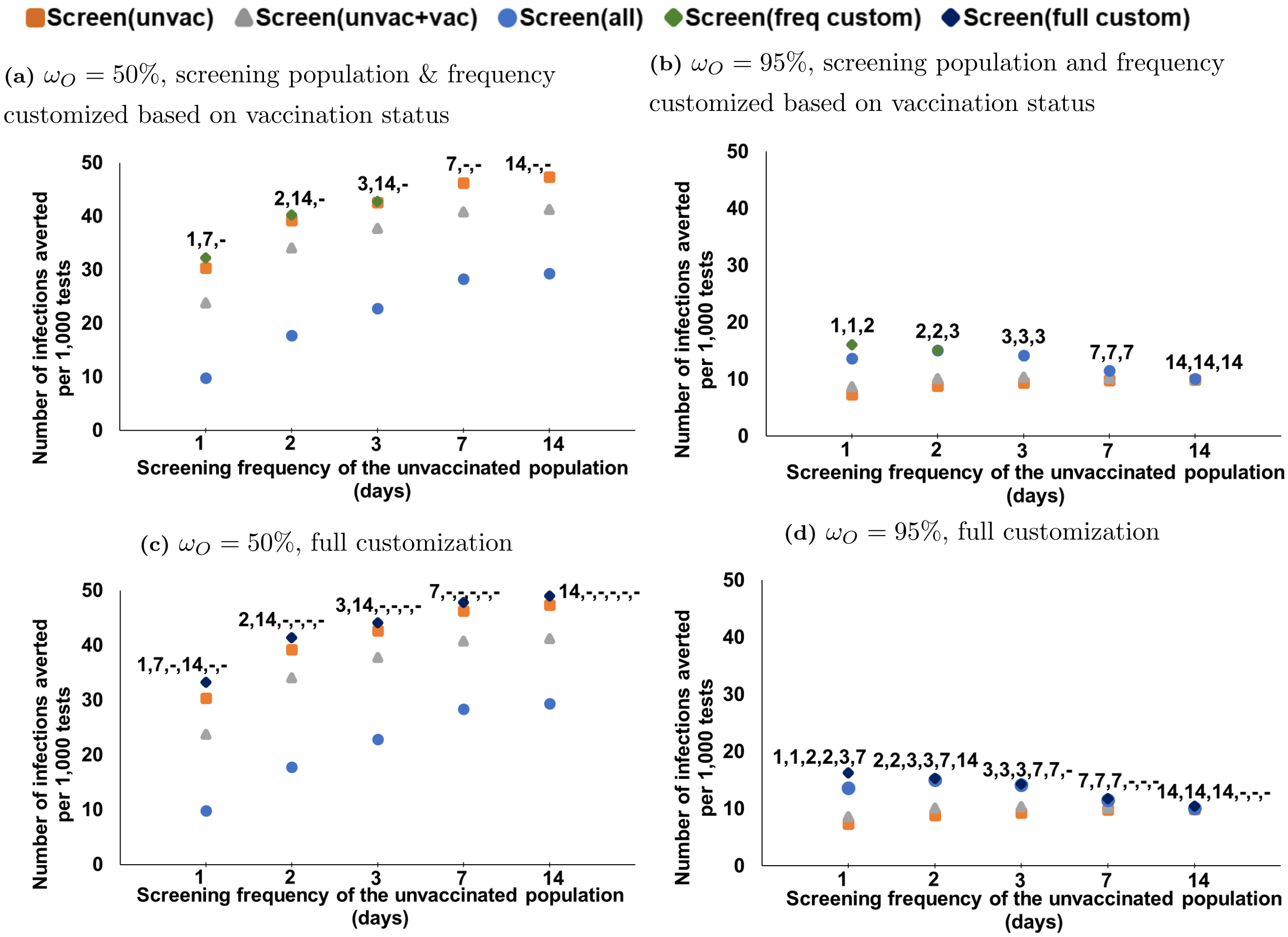

(a) $\omega_O = 50\%$, screening population & frequency customized based on vaccination status

(b) $\omega_O = 95\%$, screening population and frequency customized based on vaccination status

(c) $\omega_O = 50\%$, full customization

(d) $\omega_O = 95\%$, full customization

Supplementary Figure S4: Number of infections averted per 1,000 tests with respect to the screening frequency of the unvaccinated, for various customized screening strategies under 64% boosted, 18% vaccinated, 18% unvaccinated, best-case transmission and various Omicron proportions. (a)-(b): Screening is customized based on vaccination status only; the label represents the screening frequency for unvaccinated, vaccinated, boosted. (c)-(d): Screening is customized based on both vaccination status and faculty versus students; the label represents the screening frequency for unvaccinated students, vaccinated students, boosted students, unvaccinated faculty, vaccinated faculty, boosted faculty. ("-" indicates no screening.)

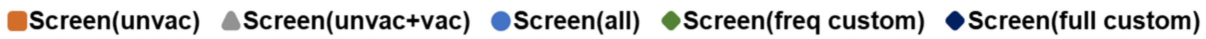

(a) $\omega_O = 50\%$, screening population & frequency customized based on vaccination status

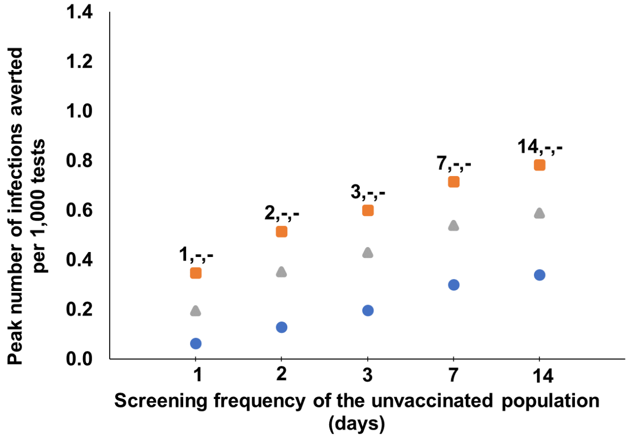

(b) $\omega_O = 95\%$, screening population & frequency customized based on vaccination status

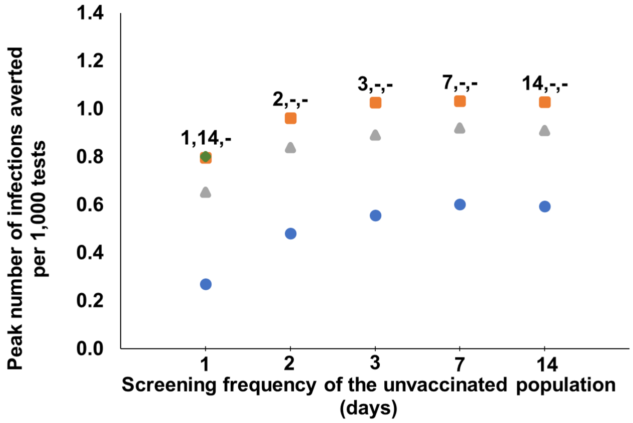

(c) $\omega_O = 50\%$, full customization

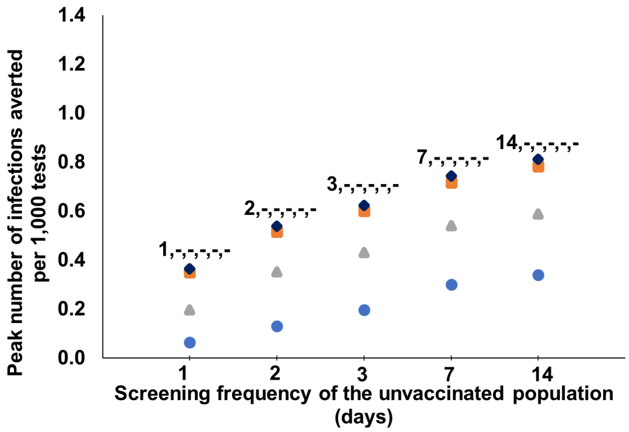

(d) $\omega_O = 95\%$, full customization

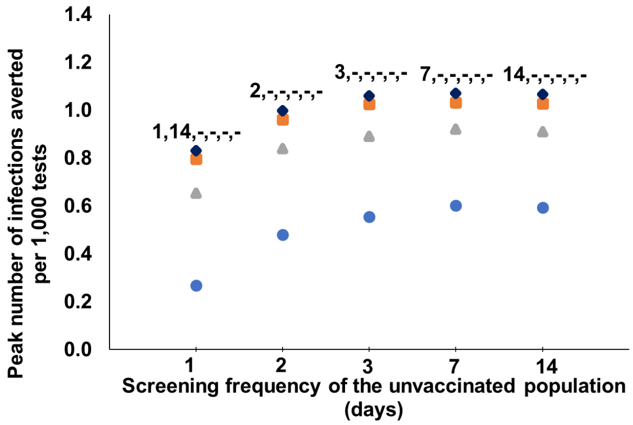

Supplementary Figure S5: Peak number of infections averted per 1,000 tests with respect to the screening frequency of the unvaccinated, for various customized screening strategies under 64% boosted, 18% vaccinated, 18% unvaccinated, best-case transmission and various Omicron proportions. (a)-(b): Screening is customized based on vaccination status only; the label represents the screening frequency for unvaccinated, vaccinated, boosted. (c)-(d): Screening is customized based on both vaccination status and faculty versus students; the label represents the screening frequency for unvaccinated students, vaccinated students, boosted students, unvaccinated faculty, vaccinated faculty, boosted faculty. ("-" indicates no screening).

References

[1] H. Barratt, M. Kirwan, and S. Shantikumar. Epidemic theory (effective & basic reproduction numbers, epidemic thresholds) & techniques for analysis of infectious disease data (construction & use of epidemic curves, generation numbers, exceptional reporting & identification of significant clusters). *Health Knowledge*, 2018.

[2] CDC. CDC updates and shortens recommended isolation and quarantine period for general population, Accessed on December 2021. `https://www.cdc.gov/media/releases/2021/s1227-isolation-quarantine-guidance.html`.

[3] CDC. Demographic trends of COVID-19 cases and deaths in the US reported to CDC, Accessed on November 2021. `https://covid.cdc.gov/covid-data-tracker/#demographics`.

[4] CNN. You asked, we're answering: Your top questions about COVID-19 and vaccines, Accessed on January 2022. `https://www.cnn.com/interactive/2020/health/coronavirus-questions-answers/#are-fully-vaccinated-people-protected-against-the-omicron-variant-how-effective-ar`

[5] COVID-Net. Laboratory-confirmed COVID-19-associated hospitalizations, Accessed on November 2021. `https://gis.cdc.gov/grasp/COVIDNet/COVID19_3.html`.

[6] Y. Liu and J. Rocklöv. The reproductive number of the delta variant of SARS-CoV-2 is far higher compared to the ancestral SARS-CoV-2 virus. *Journal of travel medicine*, 2021.

[7] F. P. Lyngse, L. H. Mortensen, M. J. Denwood, L. E. Christiansen, C. H. Møller, R. L. Skov, K. Spiess, A. Fomsgaard, R. Lassauniere, M. Rasmussen, et al. SARS-CoV-2 Omicron VOC transmission in Danish households. *MedRxiv*, 2021.

[8] P. Poletti, M. Tirani, D. Cereda, F. Trentini, G. Guzzetta, G. Sabatino, V. Marziano, A. Castrofino, F. Grosso, G. Del Castillo, et al. Association of age with likelihood of developing symptoms and critical disease among close contacts exposed to patients with confirmed SARS-CoV-2 infection in Italy. *JAMA network open*, 4(3):e211085–e211085, 2021.

[9] M. J. Rabil, S. Tunc, D. R. Bish, and E. K. Bish. Benefits of integrated screening and vaccination for infection control. *PloS ONE*, 17(4):e0267388, 2022. doi: https://doi.org/10.1371/journal.pone.0267388.

[10] Scientific American. The risk of vaccinated COVID transmission is not low, Accessed on December 2021. https://www.scientificamerican.com/article/the-risk-of-vaccinated-covid-transmission-is-not-low/.

[11] Statista. Number of Coronavirus disease 2019 (COVID-19) deaths in the U.S. as of November 24, 2021, by age*, Accessed on November 2021. https://www.statista.com/statistics/1191568/reported-deaths-from-covid-by-age-us/.

[12] Statista. Total number of cases of COVID-19 in the United States as of November 24, 2021, by age group, Accessed on November 2021. https://www.statista.com/statistics/1254271/us-total-number-of-covid-cases-by-age-group/.

[13] W. T. Zha, F. R. Pang, N. Zhou, B. Wu, Y. Liu, Y. B. Du, X. Q. Hong, and Y. Lv. Research about the optimal strategies for prevention and control of varicella outbreak in a school in a central city of China: Based on an SEIR dynamic model. *Epidemiology & Infection*, 148, 2020.
